# TRIM47 promotes ovarian cancer cell proliferation, migration, and invasion by activating STAT3 signaling

**DOI:** 10.1016/j.clinsp.2022.100122

**Published:** 2022-10-23

**Authors:** Xi Wang, Yu Fu, Yanyan Xing

**Affiliations:** a Department of Emergency and Critical Care Medicine, Jinshan Hospital, Fudan University, Shanghai, China; b Department of Obstetrics and Gynecology, Jinshan Hospital, Fudan University, Shanghai, China

**Keywords:** TRIM47, STAT3, Proliferation, Metastasis, Ovarian cancer

## Abstract

**Objectives:**

Tripartite Motif 47 (TRIM47) protein plays a prominent role in many cancers. This study aimed to investigate the biological roles of TRIM47 in ovarian cancer.

**Methods:**

TRIM47 was knocked down and overexpressed in ovarian cancer cell lines SKOV3 and OVCAR3, and the effects on proliferation, clone formation, apoptosis, invasion, and growth of xenograft tumors in nude mice were determined. The expression levels of the selected candidates were tested by western blotting and quantitative real-time PCR.

**Results:**

TRIM47 knockdown suppressed proliferation and encourages apoptosis of ovarian cancer cells. Similarly, TRIM47 knockdown suppressed ovarian cancer cell invasion, migration, and epithelial-mesenchymal transition. Ovarian cancer cell xenograft assays demonstrated that TRIM47 knockdown significantly inhibited tumor growth. Mechanistically, TRIM47 knockdown suppressed STAT3 phosphorylation and the expression of several downstream genes, including MCL-1, MMP2, and c-MYC. Silencing of STAT3 partially prevented TRIM47–induced tumor cell proliferation and invasion.

**Conclusion:**

The present study's findings demonstrate that by activating STAT3 signaling, TRIM47 functions as an oncogene in ovarian cancer. TRIM47, therefore, appears to be a potential target for ovarian cancer prevention and/or therapy.

## Highlights

• Knockdown of TRIM47 suppressed proliferation of ovarian cancer cells.
• Knockdown of TRIM47 inhibited ovarian cancer cell migration and invasion.
• TRIM47 promoted ovarian cancer cell proliferation and invasion via STAT3 signaling.

## Introduction

Ovarian cancer is one of the most detrimental gynecological malignancies. In 2020 alone, approximately 313,959 new patients and 207,252 ovarian cancer deaths were reported.^1^ More than 70% of ovarian cancer cases are detected in the mid and late stages.^2^ Despite advancements in surgical and chemotherapeutic options, many patients experience recurrence and metastasis within five years. Although ovarian cancer has been studied extensively over the past several decades, further analyses are needed to better elucidate its pathogenesis, identify more valuable predictors, and develop more targeted therapies.

The Tripartite Motif (TRIM) family of proteins are evolutionarily conserved proteins that share a common N-terminal ‘Really Interesting New Gene’ (RING) finger domain, followed by 1 or 2 B-box domains and coiled-coil sequences.^3^ Many TRIM proteins function as RING-finger E3 ubiquitin ligases. Recent evidence has confirmed that aberrant TRIM expression correlates with various human cancers, including ovarian cancer, and that the various TRIM protein family members function as oncogenes or tumor suppressors.^4^ For example, TRIM52 is upregulated in ovarian cancer, and its overexpression promotes cell proliferation and invasion via the NF-κB pathway.^5^ TRIM50 functions as a novel Src suppressor and promotes ovarian cancer progression.^6^ TRIM47 is localized at 17q25.1, a region that is frequently amplified in numerous other cancers.^7^ TRIM47 upregulation has been associated with human colorectal cancer, renal cell carcinoma, pancreatic cancer, and breast cancer.^7^, ^8^, ^9^, ^10^ However, its functions and mechanisms in ovarian cancer remain largely unexplored.

The authors here report that TRIM47 knockdown suppressed proliferation and invasion and promoted apoptosis of ovarian cancer cells, whereas TRIM47 overexpression enhanced cell proliferation and invasion. In addition, TRIM47 enhanced STAT3 phosphorylation, and the knockdown of STAT3 attenuated the oncogenic role of TRIM47 in ovarian cancer. These observations shed light on the mechanism by which TRIM47 promotes ovarian cancer.

## Materials and methods

### Reagents, antibodies, and cells

Antibodies against TRIM47, β-actin, Bax, Bcl-2, E-cadherin, vimentin, and STAT3 were purchased from Proteintech (Wuhan, China). Antibodies against phospho-STAT3 (Tyr-705) were obtained from Cell Signaling Technology (Danvers, MA, USA). The secondary antibody for western blotting was purchased from Servicebio (Wuhan, China). EdU proliferation supplies, CCK-8 kit, and enhanced chemiluminescent reagent were acquired from the Boyetime (Shanghai, China). TRIzol® reagent was purchased from Invitrogen (Grand Island, NY, USA). The human ovarian cancer cell lines SKOV3 and OVCAR3 were purchased from the Chinese Academy of Sciences Cell Bank (Shanghai, China) and grown in RPMI-1640 medium (Keygen Biotech, Nanjing, China) supplemented with 10% FBS (Gibco, Grand Island, NY, USA) in a humidified environment with 5% CO_2_ at 37°C.

### Plasmid, lentivirus infection, and siRNA

shRNA sequences for TRIM47 (shTRIM47) and Negative Control shRNA (NC) were cloned into the lentiviral vector pLKO.1. Virus particles were harvested 48h after lentiviral vector transfection alongside the packaging plasmids in HEK293T cells using Lipofectamine 2000. Ovarian cancer cells were infected with lentivirus-transducing units in the presence of 5 mg/mL polybrene. The TRIM47 coding sequence was cloned into pcDNA3.1 (+) to generate a TRIM47 overexpression vector. STAT3 siRNA was procured from GeneChem (Shanghai, China). Cells were collected 48h after plasmid or siRNA transfection.

### Western blotting

Western blotting was performed as previously described.^11^ Briefly, cells or tissues were lysed on ice in RIPA buffer containing protease inhibitors. The cell lysates were subjected to sodium dodecyl sulfate-polyacrylamide gel electrophoresis and then transferred to PVDF membranes. After blocking, the membranes were incubated with primary antibodies overnight at 4°C, rinsed three times with TBST before incubation with horseradish peroxidase-conjugated secondary antibodies, followed by three washes, and the antibody-labeled protein bands were detected by Enhanced Chemiluminescence (ECL). Densitometric analysis of the ECL signal was performed using ImageJ software.

### Quantitative real-time PCR (qPCR)

cDNA synthesis was performed using a PrimeScript™ reverse transcriptase reagent kit (Takara, Japan), and total RNA was isolated from tissues or cells using TRIzol® reagent. The mRNA levels of E-cadherin, vimentin, Mcl-1, MMP2, and c-Myc were determined by qPCR using SYBR Green (Thermo Fisher Scientific, Waltham, MA, USA) on an ABI 7900 instrument (Applied Biosystems, Foster City, CA, USA) with GAPDH as an internal control. The fold-change in gene expression normalized by the internal control was estimated by the 2^−△△CT^ method and individual data represent triplicate measurements.

### Cell proliferation assays

Cell proliferation was measured using a 5-Ethynyl-2′-Deoxyuridine (EdU) proliferation detection kit according to the manufacturer's instructions. Briefly, the cells were pretreated with EdU for 2h before being stained with DAPI. Images of EdU-positive cells were captured using a fluorescence microscope (Olympus, Tokyo, Japan). For the Cell Counting Kit-8 (CCK-8) test, transfected cells were plated at 2 × 10^3^ cells in 96-well plates and cultured for 24h, 48h, 72h, and 96h. Then, 20 μL of CCK-8 reagent was added to each well and cultured for a further two hours. A standard microplate reader was used to measure the absorbance at 450 nm. For the colony formation assay, 1000 cells were plated in a 6-well plate and maintained in RPMI-1640 medium containing 10% FBS for two weeks. After methanol fixation, the colonies were stained with 0.1% crystal violet for 15 min before being counted. All assays were performed in triplicate.

### Transwell migration and invasion analyses

For the migration test, 1 × 10^5^ cells in 200 μL of RPMI-1640 medium without serum were placed in Boyden chambers (Corning Life Science, Corning, NY, USA). Similar cell quantities were placed in Boyden chambers covered with 150 mg Matrigel® (BD Biosciences, San Jose, CA, USA) for the invasion test. Respective sections were subsequently infused into 24-well plates before 24h of incubation in RPMI-1640 medium supplemented with 10% FBS. Cells that remained on the upper surface of the membrane were removed, while those adhered to the lower surface were fixed, stained for 1h in a solution containing 0.05% crystal violet, and then counted using an Olympus microscope to determine their respective relative numbers. All results are presented as the mean of three separate analyses.

### Cell apoptosis measurement

To measure cell apoptosis, 5 × 10^5^ cells were fixed in ice-cold 70% ethanol, followed by incubation with the testing reagents of the Annexin V-FITC apoptosis kit (Boyetime) according to the manufacturer's guidelines. The fluorescence signal was detected using a BD FACSCanto™ flow cytometer and analyzed using FlowJo™ software.

### Bioinformatics analysis of the TCGA database

The TCGA database outcomes were primarily investigated using web-based gene expression profiling interactive analysis (GEPIA; http://gepia.cancer-pku.cn/).^12^ GEPIA generally provides rapid and cutting-edge applications established on the TCGA data and supports various functions, including patient survival analysis, differential expression analysis, and correlation analysis.

### Xenograft tumor model

All the animal experimental procedures were approved by the Institutional Animal Care and Use Committee of Jinshan Hospital (Ethical permission number: Y2022-050). Six-week-old female BALB/c nude mice were purchased from the SLAC Laboratory Animal Co. Ltd. (Shanghai, China). Subcutaneous xenografts were established by subcutaneous injection of 2 × 10^6^ TRIM47 knockdown or control SKOV3 cells into the flanks of mice. Five weeks later, the mice were examined for the presence of tumors, and the tumors were harvested after the animals were culled. Tumor dimensions were determined by measuring the tumor width and length using calipers, and volumes were estimated using the following formula: (A × B^2^) × 0.5, where A is the length and B is the width of the tumor. The wet weight of each tumor was also determined.

### Immunohistochemistry (IHC) and TUNEL assay

IHC was performed according to a standard method, as described previously.^13^ Briefly, paraffin-embedded samples were cut into 4 µm sections, deparaffinized, rehydrated and then exposed to a heat-induced homolog repossession stage in 0.01 M sodium citrate (Ph 6.0). Endogenous peroxidase activity was blocked by incubation in 3% hydrogen peroxide in distilled water for 25 min. The sections were then incubated with 3% serum for 30 min. Subsequently, the samples were incubated overnight at 4°C with rabbit polyclonal Ki67 antibody. Finally, the sections were stained and counterstained using DAB and hematoxylin, respectively. TUNEL assay was performed using an in situ cell death detection kit (Roche, Tokyo, Japan), and the number of positive cells was estimated. The apoptotic index was calculated as the (apoptotic cell number)/(total cell number) ratio.

### Statistical analysis

All data are presented as means ± the Standard Deviation (SD). Data between two groups were compared quantitatively using Student's *t*-test, while one-way analysis of variance (ANOVA) was used for multiple groups. Statistical significance was set at p < 0.05. All estimations were performed using the GraphPad Prism software (version 5.0).

## Results

### Knockdown of TRIM47 suppressed proliferation and promotes apoptosis of ovarian cancer cells

**Fig. 1:**
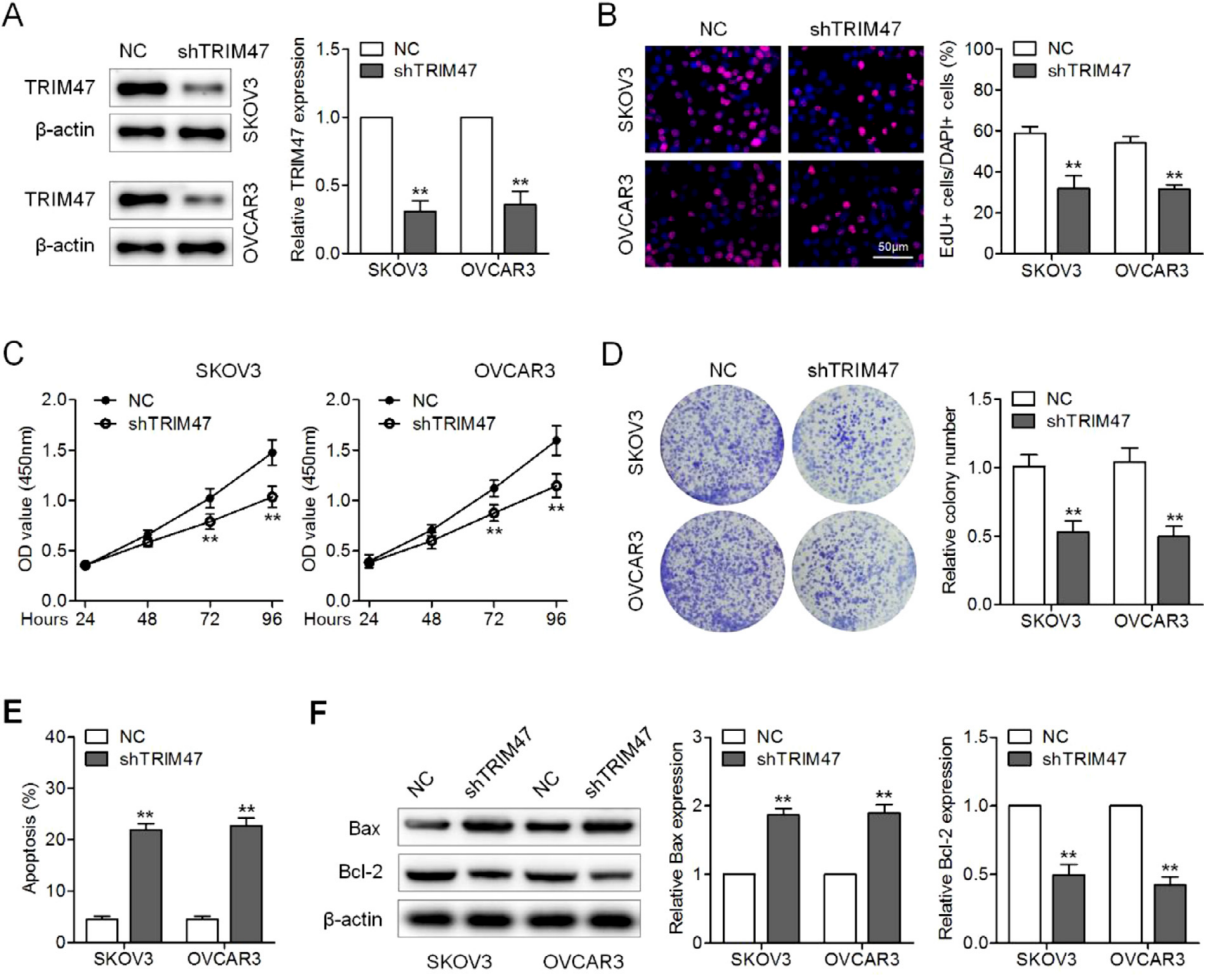
Knockdown of TRIM47 suppressed proliferation and increased apoptosis in ovarian cancer cells. (A) SKOV3 and OVCAR3 cells were transduced with lentivirus expressing shRNA against TRIM47 (shTRIM47) or Negative Control (NC); TRIM47 protein expression was analyzed by western blotting. (B) Fluorescence microscopy images (left) and quantification (right) of EdU-positive cells in the infected SKOV3 and OVCAR3 cells. Scale bar: 50 μm. (C) Cell viability was examined in the infected cells by CCK-8 assay. (D) Low-magnification images (left) and quantification (right) of SKOV3 and OVCAR3 cell colony formation. (E) Annexin V/propidium iodide double-staining flow cytometry was performed to determine cell apoptosis. (F) Western blots (left) were quantified to determine the levels of the apoptosis markers Bax (middle) and Bcl-2 (right) in the infected cells. ^⁎⁎^p < 0.01.

To explore the role of TRIM47 in ovarian cancer tumorigenesis, the authors first performed loss-of-function assays by silencing TRIM47 with lentiviruses expressing shRNA against TRIM47 in the ovarian cancer cell lines OVCAR3 and SKOV3. Western blotting verified the successful knockdown of TRIM47 in these cells (Fig. 1A). EdU incorporation analysis revealed fewer proliferating cells in the TRIM47 knockdown group than in the control group (Fig. 1B). Similarly, the results of CCK8 and colony formation assays demonstrated that TRIM47 knockdown significantly inhibited cell viability and colony formation compared to the control group (Fig. 1C and D). Annexin V-FITC/PI staining was used to analyze apoptosis of the ovarian cancer cell lines. The flow cytometry results revealed increased apoptosis in TRIM47 knockdown cells than in the control cells (Fig. 1E). Enhanced Bax and reduced Bcl-2 expression were consistently observed in the TRIM47 knockdown cells (Fig. 1F). These observations indicate that reduction of TRIM47 expression suppresses proliferation and promotes apoptosis in ovarian cancer cells.

### Knockdown of TRIM47 inhibited ovarian cancer cell migration and invasion

**Fig. 2:**
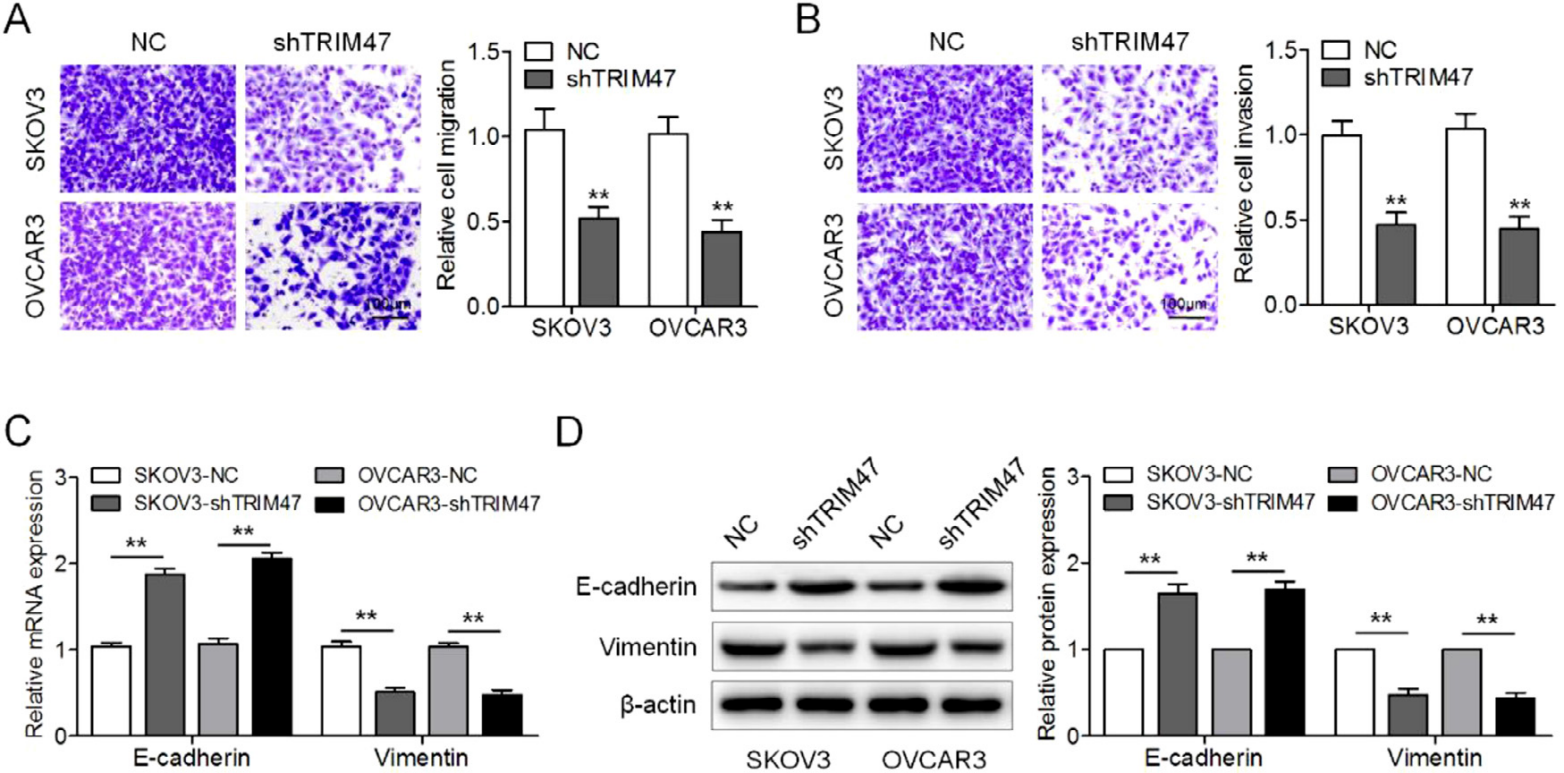
TRIM47 knockdown inhibited ovarian cancer cell migration, invasion, and EMT. (A) Imaging of the Transwell® migration membranes (left) and quantification of the number of cells (right) showed that TRIM47 knockdown reduced SKOV3 and OVCAR3 cell migration. Scale bar: 100 μm. (B) Imaging of the Transwell® invasion assay membrane (left) and quantification of the number of cells (right) showed that TRIM47 knockdown reduced SKOV3 and OVCAR3 cells invasion. Scale bar: 100 μm. (C) RT-qPCR quantification of the expression of EMT-associated proteins E-cadherin and vimentin mRNAs in SKOV3 and OVCAR3 cells. (D) Western blotting of EMT-associated proteins E-cadherin and vimentin in SKOV3 and OVCAR3 cells. ^⁎⁎^p < 0.01.

Next, the authors examined the effect of TRIM47 knockdown on ovarian cancer cell migration and invasion. In keeping with the Transwell® migration test results, the numbers of migrating SKOV3 and OVCAR3 cells were reduced after TRIM47 knockdown (Fig. 2A). In addition, the Transwell® invasion assay showed that TRIM47 knockdown induced a decrease in the number of invasive cells (Fig. 2B). To further determine whether TRIM47 regulates the EMT process in ovarian cancer cells, the authors assayed expression of the epithelial marker E-cadherin and the mesenchymal marker vimentin by RT-qPCR and western blotting. TRIM47 knockdown resulted in the upregulation of E-cadherin, while the level of vimentin was reduced in both SKOV3 and OVCAR3 cells (Fig. 2C and D). These observations indicate that TRIM47 knockdown suppresses ovarian cancer cell migration and invasion.

### Knockdown of TRIM47 suppressed STAT3 signaling

**Fig. 3:**
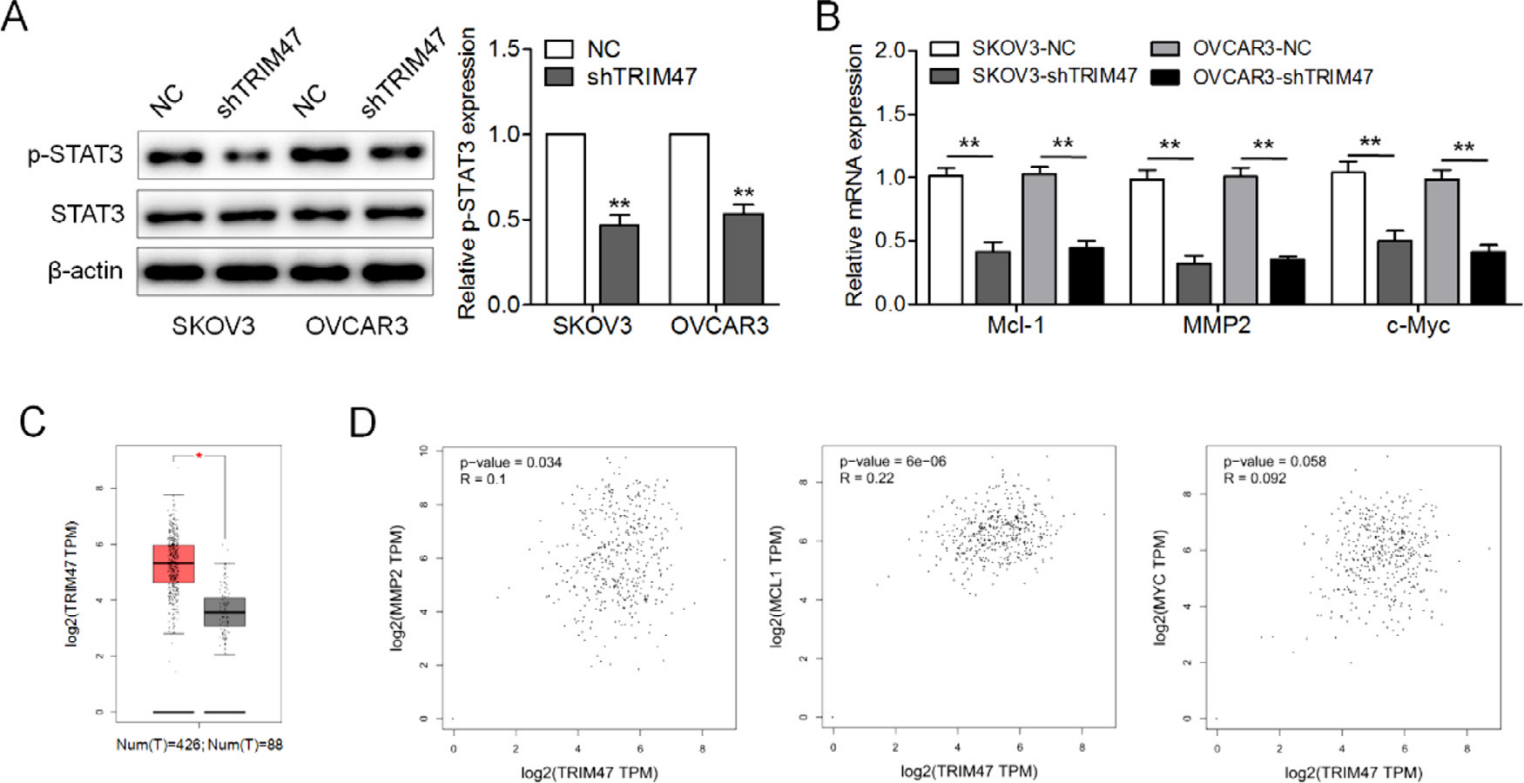
TRIM47 knockdown suppressed the STAT3 signaling pathway in ovarian cancer. (A) p-STAT3 and STAT3 levels in TRIM47 knockdown SKOV3 and OVCAR3 cells were determined by western blotting. (B) RT-qPCR measurement the expression of the STAT3 target genes MCL1, MMP2, and c-MYC. (C) TRIM47 mRNA expression data corresponding to ovarian cancer tissues (n = 426) and normal tissues (n = 88) were retrieved from a GEPIA database. (D) Spearman's correlation analyses between TRIM47 expression and Mcl-1, MMP2, and c-Myc, respectively. ^⁎⁎^p < 0.01.

The STAT3 signaling pathway is generally thought to be involved in cell proliferation, survival, motility, and invasion.^14^ To explore how TRIM47 affects this signaling pathway, the levels of STAT3 and phosphorylated STAT3 (p-STAT3) were evaluated by western blotting. TRIM47 knockdown in ovarian cancer cells resulted in a substantial reduction in p-STAT3 levels (Fig. 3A). The authors also examined the three STAT3 target genes MCL1, MMP2, and c-MYC, and we found that the expression of these genes was significantly reduced in SKOV3 and OVCAR3 cells following TRIM47 knockdown. This suggests that TRIM47 knockdown reduces the transcriptional activity of STAT3 downstream signaling (Fig. 3B). The authors also used GEPIA to analyze the mRNA levels of TRIM47 and STAT3 target genes in ovarian cancer tissues. The results show that the level of TRIM47 mRNA in ovarian cancer specimens was significantly higher than that in normal tissues (Fig. 3C). Moreover, TRIM47 expression was positively correlated with MMP2 and Mcl-1 expression (Fig. 3D).

### TRIM47 promoted ovarian cancer cell proliferation and invasion by activating STAT3 signaling

**Fig. 4:**
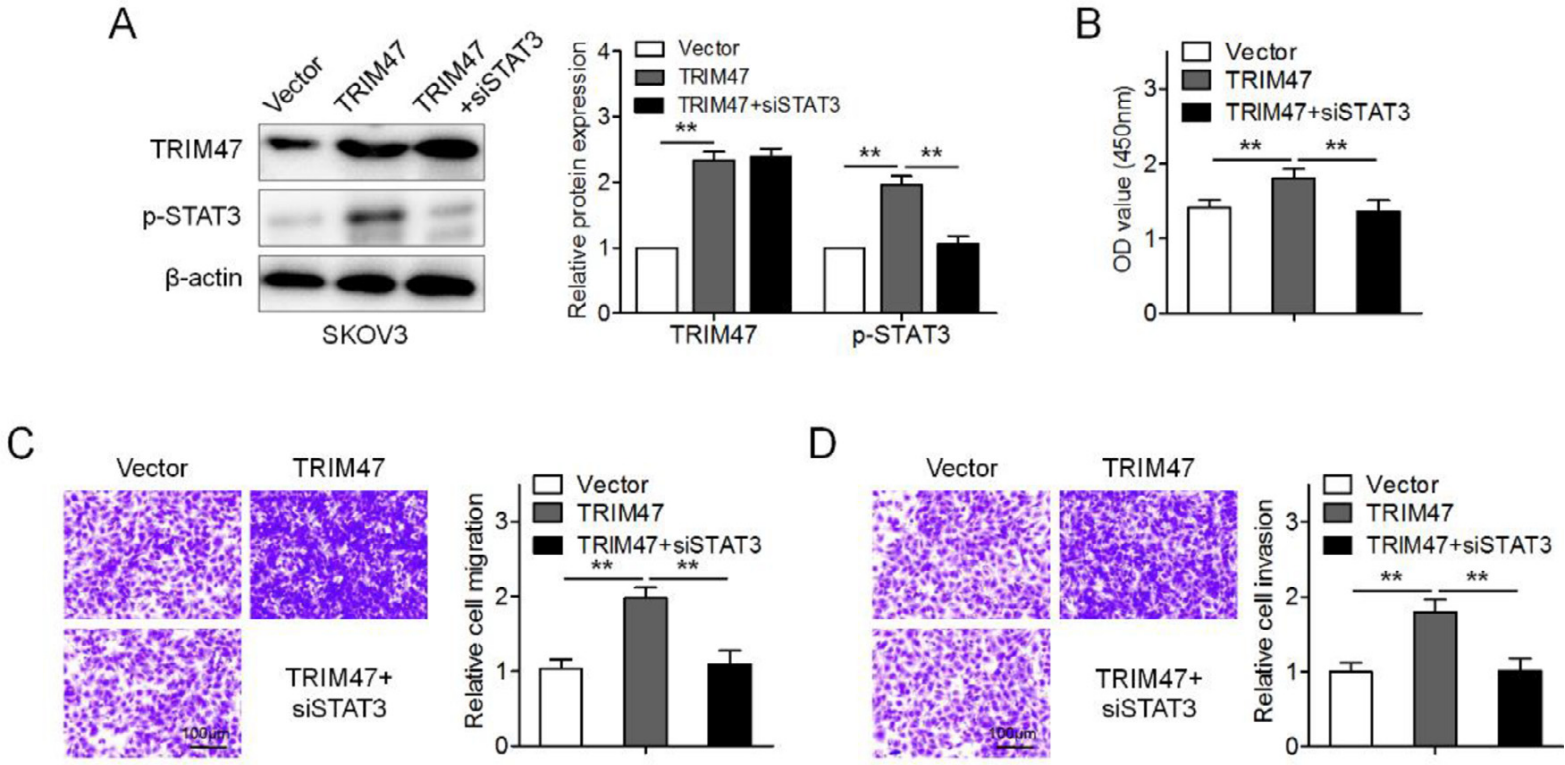
TRIM47 promoted cell proliferation and invasion by activating the STAT3 signaling pathway. (A) SKOV3 cells were transfected with TRIM47 plasmids, as well as co-transfected with TRIM47 plasmids and siRNA against STAT3 (TRIM47+siSTAT3), respectively, and western blotting was used to determine TRIM47 and STAT3 levels. (B) CCK-8 assay for the transfected SKOV3 cells. (C) Transwell® migration assay for the marked cells. Scale bar: 100 μm. (D) Transwell® invasion assay for the denoted cells. Scale bar: 100 μm. ^⁎⁎^p < 0.01.

To further determine whether TRIM47 functions in ovarian cancer by activating STAT3 signaling, SKOV3 cells were transfected with TRIM47 plasmids. TRIM47 overexpression enhanced the levels of p-STAT3 and promoted cell proliferation, migration, and invasion (Fig. 4A–D). When STAT3 siRNA was co-transfected into SKOV3 cells, p-STAT3 expression significantly diminished in the TRIM47 overexpression cells (Fig. 4A). Interestingly, STAT3 knockdown significantly reduced TRIM47-induced cell proliferation, migration, and invasion (Figs. 4B–D). These observations suggest that TRIM47 functions as an oncogenic factor in ovarian cancer partially through STAT3.

### Down-regulation of TRIM47 suppressed tumorigenesis in a xenograft model

**Fig. 5:**
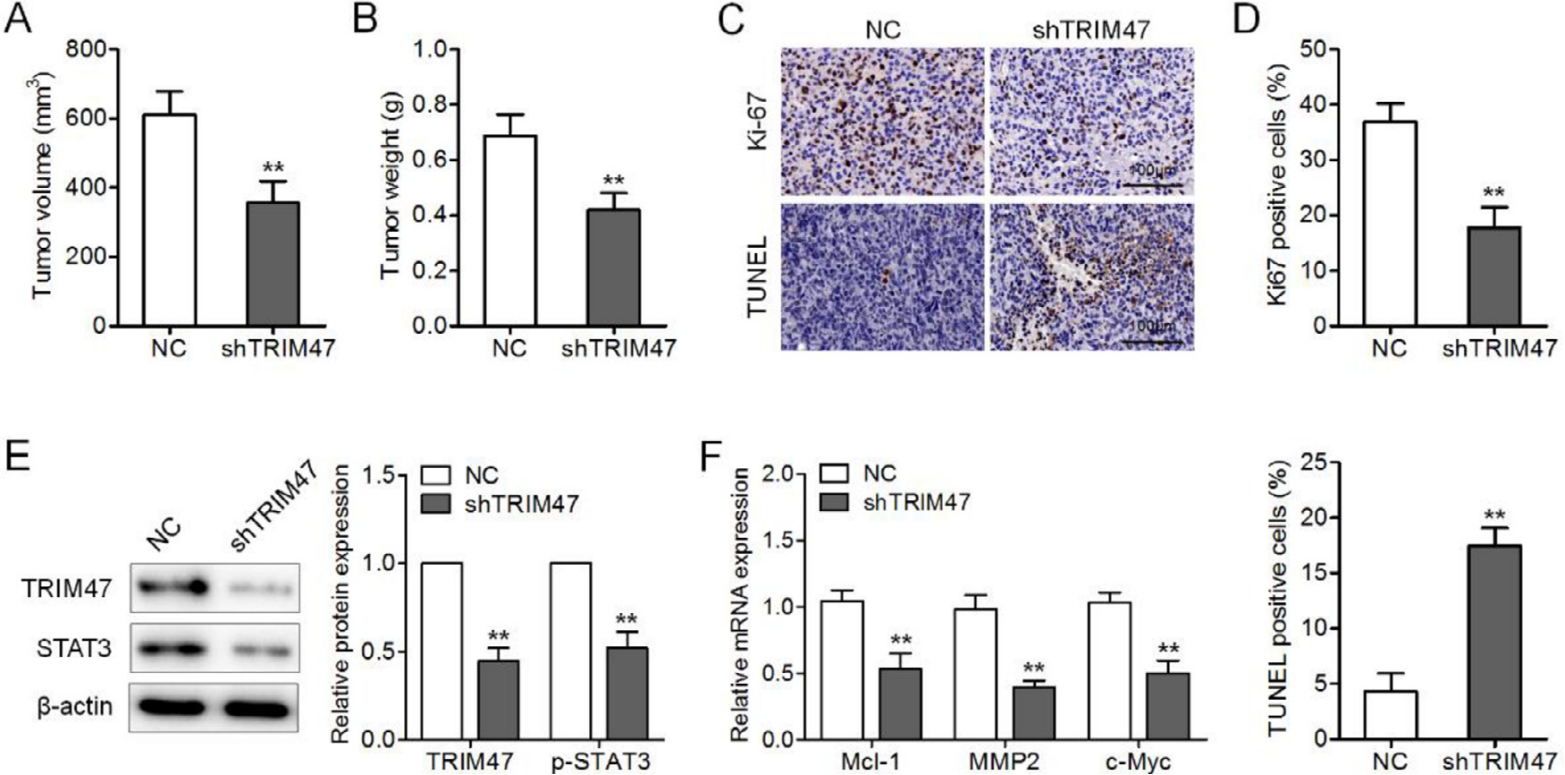
TRIM47 knockdown suppressed ovarian cancer growth *in vivo*. TRIM47 knockdown or NC SKOV3 cells were injected subcutaneously into nude mice, and those mice were carefully monitored for tumor growth for five weeks. (A) The proportional volumes of the xenograft tumors. (B) Proportional weights of the xenograft tumors. (C, D) Ki-67 and TUNEL assay IHC in the xenograft tumor tissues. Scale bar: 100 μm. (E) Levels of TRIM47 and p-STAT3 protein in the tumors as determined by western blotting. (F) RT-qPCR analysis of Mcl-1, MMP2, and c-Myc mRNA levels in the tumors. ^⁎⁎^p < 0.01.

Finally, the authors generated a xenograft tumor model by intravenous injection of TRIM47 knockdown SKOV3 cells into nude mice to further assess the influence of TRIM47 on tumor growth *in vivo*. When the tumors were harvested, the weights and volumes of the tumors in the TRIM47 knockdown group were lower than those in the control groups (Fig. 5A and B). In addition, the number of Ki-67-positive tumor cells was lower, whereas the number of TUNEL-positive cells was higher in the TRIM47 knockdown group than in the controls (Fig. 5C and D). Western blotting further confirmed the TRIM47 knockdown and reduced p-STAT3 protein levels in the TRIM47 knockdown group (Fig. 5E). Mcl-1, MMP2, and c-Myc mRNA levels were also reduced in the xenograft tumor tissues with TRIM47 knockdown (Fig. 5F). These observations demonstrate that TRIM47 knockdown reduced tumor growth *in vivo*.

## Discussion

An increasing number of studies have shown that TRIM protein cancer either promotes or entirely or inhibits carcinogenesis.^4^ This study analyzed TRIM47 biological function in human ovarian cancer and yielded experimental evidence that TRIM47 upregulation in ovarian cancer promotes cell proliferation, migration, and invasion by activating STAT3.

TRIM47 is frequently overexpressed in human cancers and promotes tumor cell proliferation and metastasis. For example, Liang et al. reported that TRIM47 overexpression is common and associated with a poor prognosis of colorectal cancer in patients, resulting in increased proliferation and metastasis of colorectal cancer cells.^7^ Li et al. reported that TRIM47 is upregulated in pancreatic cancer, and its overexpression promotes aerobic glycolysis and tumor growth.^9^ Furthermore, Azuma et al. showed that TRIM47 was a poor prognostic factor in breast cancer patients who underwent endocrine therapy with tamoxifen.^10^ Consistent with these findings, the authors confirmed an oncogenic role of TRIM47 in ovarian cancer cells using *in vitro* and *in vivo* experiments. The present results indicate that TRIM47 knockdown reduced cell proliferation, migration, invasion, and EMT, whereas its overexpression enhanced proliferation, migration, and invasion. The xenografts of the TRIM47 knockdown group grew slower and its tumor weight was also smaller than that in the control group. Additionally, flow cytometry and TUNEL assays both demonstrated that TRIM47 overexpression reduced ovarian cancer cell apoptosis. Conversely, TRIM47 knockdown resulted in a significant increase in Bax and a decrease in Bcl-2 levels.

Signal transducers such as STAT3 in various cancer classifications, including ovarian cancer, are aberrantly activated by tyrosine phosphorylation.^15^ Activated STAT3 has been shown to increase the proliferation, metastasis, and chemoresistance of ovarian cancer cells, as well as angiogenesis.^16^ At the clinical level, there is increasing evidence that activation of the STAT3 pathway significantly correlates with reduced survival of rare ovarian cancers, thereby highlighting the importance of STAT3 as a prospective therapeutic target for cancer treatment.^15^ Many TRIM proteins have been reported to activate the STAT3 pathway, thereby promoting cancer progression. For example, TRIM32 positively regulates p-STAT3 levels in lung cancer,^17^ and TRIM52 promotes SHP2 ubiquitination, consequently inactivating STAT3 signaling in colorectal cancer.^18^ Here, the authors found that TRIM47 knockdown reduced STAT3 phosphorylation at Tyr705 in both cultured and xenografted ovarian cancer cells, followed by reduced expression of the STAT3 target genes MCL-1, MMP2, and c-MYC. In contrast, TRIM47 overexpression enhanced the level of p-STAT3. Functional assays showed that TRIM47 overexpression stimulated cell proliferation, migration, and invasion. However, STAT3 knockdown abolished these effects, suggesting that TRIM47 is an oncogenic factor in ovarian cancer that at least in part exerts its activity through STAT3. Additional research is required to clarify the detailed mechanism by which TRIM47 activates the STAT3 pathway.

Nevertheless, it is undeniable that there are several potential limitations that should be taken into consideration when interpreting the results of the current analysis. Although TCGA analysis revealed that TRIM47 was highly expressed in ovarian cancer, additional prospective clinical trials with a large population are still warranted to validate the upregulation of TRIM47 in ovarian cancer and evaluate its clinical significance.

**Fig. 6:**
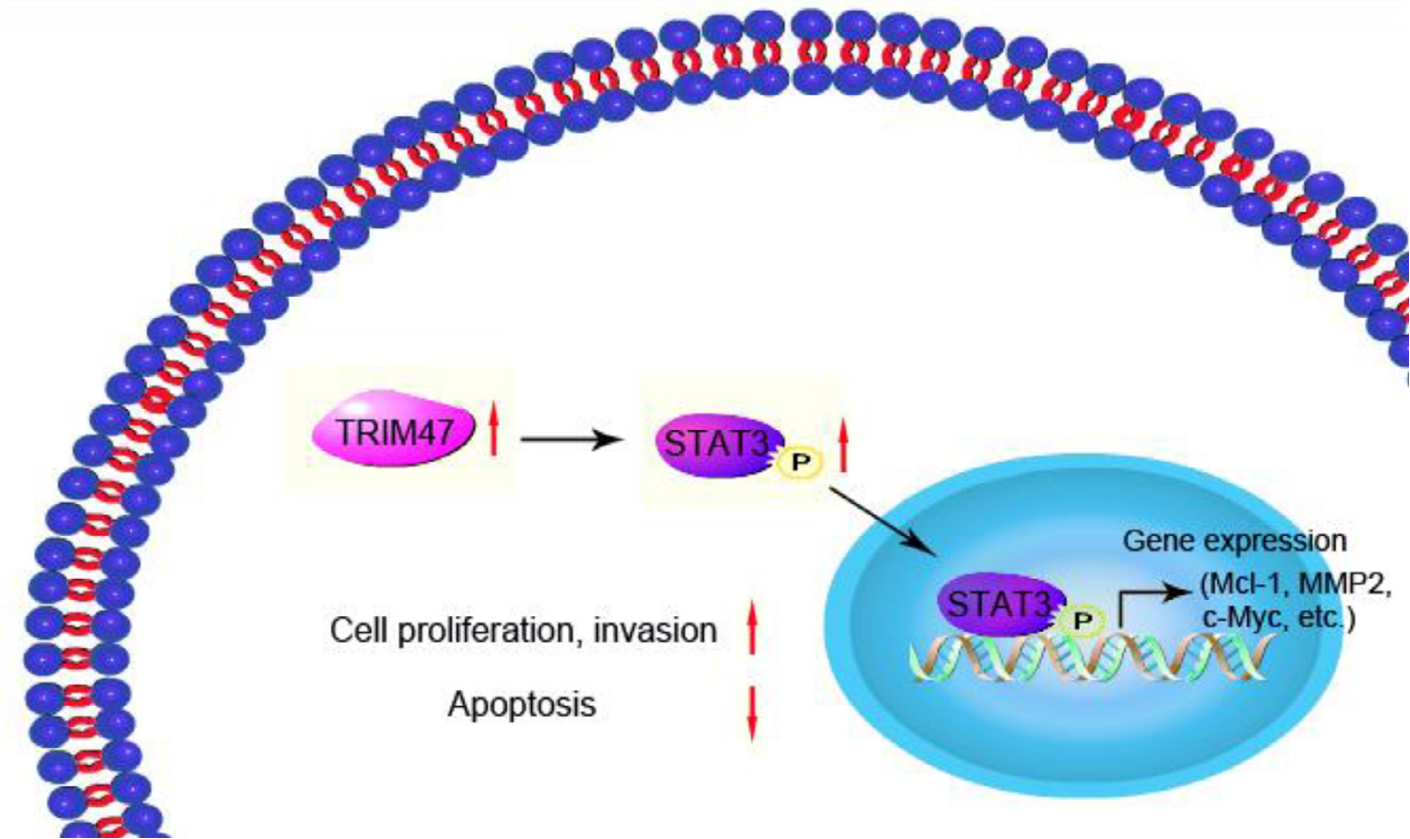
TRIM47 positively regulates STAT3 signaling, thereby promoting ovarian cancer cell proliferation and invasion.

In conclusion, the present study shows that TRIM47 positively regulates STAT3 signaling, thereby promoting tumor growth and progression (Fig. 6). These observations indicate a possible function for TRIM47 in ovarian cancer carcinogenesis, which may hence provide a therapeutic target for treating ovarian cancer.

## Authors' contributions

XW, YF and YX conceived and designed the study, and acquired, analyzed, and interpreted the data. XW and YX wrote and revised the manuscript. All of the authors agreed to be accountable for all aspects of the work in ensuring that questions related to the accuracy and integrity of any part of the work are appropriately investigated and resolved. All authors confirm the authenticity of all the raw data, and read and approved the final manuscript.

## Funding

This study was supported by the Science and Technology Innovation Fund of Jinshan District of Shanghai (Grant No. 2022-WS-11).

## Ethics approval

Animal experiments complied with the ARRIVE guidelines and were carried out in accordance with the National Research Council's Guide for the Care and Use of Laboratory Animals, which was approved by the Institutional Animal Care and Use Committee of Jinshan Hospital (Shanghai, China).

## Data availability statement

The datasets used and/or analyzed during the current study are available from the corresponding author upon reasonable request.

## Conflicts of interest

The authors declare no conflicts of interest.

## References

[1] Sung H, Ferlay J, Siegel RL, Laversanne M, Soerjomataram I, Jemal A (2021). Global cancer statistics 2020: GLOBOCAN estimates of incidence and mortality worldwide for 36 cancers in 185 countries. CA Cancer J Clin.

[2] Ning X, Shi G, Ren S, Liu S, Ding J, Zhang R (2022). GBAS regulates the proliferation and metastasis of ovarian cancer cells by combining with eEF1A1. Oncologist.

[3] Ozato K, Shin DM, Chang TH, Morse HC (2008). TRIM family proteins and their emerging roles in innate immunity. Nat Rev Immunol.

[4] Zhao G, Liu C, Wen X, Luan G, Xie L, Guo X. (2021). The translational values of TRIM family in pan-cancers: From functions and mechanisms to clinics. Pharmacol Ther.

[5] Yang W, Liu L, Li C, Luo N, Chen R, Li L (2018). TRIM52 plays an oncogenic role in ovarian cancer associated with NF-kB pathway. Cell Death Dis.

[6] Qiu Y, Liu P, Ma X, Ma X, Zhu L, Lin Y (2019). TRIM50 acts as a novel Src suppressor and inhibits ovarian cancer progression. Biochim Biophys Acta Mol Cell Res.

[7] Liang Q, Tang C, Tang M, Zhang Q, Gao Y, Ge Z. (2019). TRIM47 is up-regulated in colorectal cancer, promoting ubiquitination and degradation of SMAD4. J Exp Clin Cancer Res.

[8] Chen JX, Xu D, Cao JW, Zuo L, Han ZT, Tian YJ (2021). TRIM47 promotes malignant progression of renal cell carcinoma by degrading P53 through ubiquitination. Cancer Cell Int.

[9] Li L, Yu Y, Zhang Z, Guo Y, Yin T, Wu H (2021). TRIM47 accelerates aerobic glycolysis and tumor progression through regulating ubiquitination of FBP1 in pancreatic cancer. Pharmacol Res.

[10] Azuma K, Ikeda K, Suzuki T, Aogi K, Horie-Inoue K, Inoue S. (2021). TRIM47 activates NF-kappaB signaling via PKC-epsilon/PKD3 stabilization and contributes to endocrine therapy resistance in breast cancer. Proc Natl Acad Sci U S A.

[11] Zhou JN, Zeng Q, Wang HY, Zhang B, Li ST, Nan X (2015). MicroRNA-125b attenuates epithelial-mesenchymal transitions and targets stem-like liver cancer cells through small mothers against decapentaplegic 2 and 4. Hepatology.

[12] Wan J, Liu H, Feng Q, Liu J, Ming L. (2018). HOXB9 promotes endometrial cancer progression by targeting E2F3. Cell Death Dis.

[13] Zhou Z, Ji Z, Wang Y, Li J, Cao H, Zhu HH (2014). TRIM59 is up-regulated in gastric tumors, promoting ubiquitination and degradation of p53. Gastroenterology.

[14] Gritsina G, Xiao F, O'Brien SW, Gabbasov R, Maglaty MA, Xu RH (2015). Targeted blockade of JAK/STAT3 signaling inhibits ovarian carcinoma growth. Mol Cancer Ther.

[15] Wu CJ, Sundararajan V, Sheu BC, Huang RY, Wei LH. (2019). Activation of STAT3 and STAT5 signaling in epithelial ovarian cancer progression: mechanism and therapeutic opportunity. Cancers (Basel).

[16] Lu T, Bankhead A, Ljungman M, Neamati N. (2019). Multi-omics profiling reveals key signaling pathways in ovarian cancer controlled by STAT3. Theranostics.

[17] Yin H, Li Z, Chen J, Hu X. (2019). Expression and the potential functions of TRIM32 in lung cancer tumorigenesis. J Cell Biochem.

[18] Pan S, Deng Y, Fu J, Zhang Y, Zhang Z, Ru X (2019). TRIM52 promotes colorectal cancer cell proliferation through the STAT3 signaling. Cancer Cell Int.

